# Supplementary material for: Weizmannia coagulans BC99 Relieves Constipation Symptoms by Regulating Inflammatory, Neurotransmitter, and Lipid Metabolic Pathways: A Randomized, Double-Blind, Placebo-Controlled Trial
Source: Foods. 2025 Feb 15;14(4):654. doi: 10.3390/foods14040654 (PMC11854163; doi:10.3390/foods14040654)
Supplement: Supplementary file 1 [file foods-14-00654-s001.zip › foods-3434325-supplementary.pdf]

Table S1 Clinical basic indicators of participants with constipation at baseline and the 8nd week.

Name	placebo (n = 45)	probiotic (n = 45)	*p*-between Groups
Age/year	35.24 ± 1.61	34.22 ± 3.84	>0.05
Woman	41（45.6%）	39（43.3%）	-
Man	4（4.4%）	6（6.7%）	-
Height/cm	165 ± 5.39	160.25 ± 5.86	>0.05
Weight/kg	48.55 ± 8.04	48.61 ± 8.97	>0.05
AST U/L			
Baseline	20.16 ± 13.88	16.67 ± 10.40	0.181 (The *p*-value between the two groups at baseline)
8 weeks	25.44 ± 18.50	17.89 ± 13.86	< 0.05 (*p*-value between the two groups after 8 weeks of intervention)
Changes from baseline p-value2) (intergroup)	5.29 ± 14.14	1.22 ± 18.94	0.251 (The p-value of 8 weeks minus baseline between the two groups)
p-value	0.130 (p value between baseline and post-intervention in the placebo group)	0.638 (p-value between baseline and post-intervention in probiotic group)	-
ALT U/L			
Baseline	27.11 ± 10.26	26.00 ± 10.84	0.620
8 weeks	26.84 ± 11.25	22.36 ± 10.88	0.0581
Changes from baseline p-value2) (intergroup)	-0.27 ± 15.88	-3.64 ± 15.39	0.310
p-value	0.910	0.116	-
UREA μmol/L			
Baseline	5.72 ± 1.13	5.58 ± 1.31	0.590
8 weeks	5.41 ± 1.04	5.16 ± 0.97	0.242
Changes from baseline p-value2) (intergroup)	-0.31 ± 0.99	-0.43 ± 1.19	0.604
p-value	0.180	0.088	-
CREA μmol/L			
Baseline	60.85 ± 10.30	62.44 ± 13.68	0.540
8 weeks	62.80 ± 9.36	61.07 ± 10.32	0.410
Changes from baseline p-value2) (intergroup)	1.96 ± 12.83	-1.37 ± 15.53	0.271
p-value	0.350	0.884	-
TC mmol/L			
Baseline	4.86 ± 0.88	4.84 ± 0.94	0.920
8 weeks	4.54 ± 0.89	4.80 ± 0.83	0.156

Changes from baseline p-value[2)] (intergroup)	-0.33 ± 1.18	-0.04 ± 0.80	0.176
p-value	0.090	0.831	-
TG mmol/L			
Baseline	1.50 ± 0.65	1.72 ± 1.66	0.411
8 weeks	1.45 ± 0.66	1.40 ± 0.83	0.752
Changes from baseline p-value[2)] (intergroup)	-0.04 ± 0.89	-0.32 ± 1.42	0.265
p-value	0.720	0.313	-
GLU mmol/L			
Baseline	4.94 ± 0.88	5.10 ± 0.95	0.410
8 weeks	4.91 ± 0.86	4.94 ± 0.62	0.850
Changes from baseline p-value[2)] (intergroup)	-0.02 ± 1.21	-0.16 ± 0.95	0.543
p-value	0.871	0.350	-
UA μmol/L			
Baseline	322.16 ± 67.62	300.56 ± 88.07	0.195
8 weeks	280.35 ± 69.82	300.51 ± 80.13	0.210
Changes from baseline p-value[2)] (intergroup)	-41.81 ± 69.94	-0.05 ± 99.35	< 0.05
p-value	0.005	0.998	-
WBC 10^9/L			
Baseline	5.11 ± 1.42	5.08 ± 1.16	0.912
8 weeks	5.09 ± 1.58	5.12 ± 1.36	0.923
Changes from baseline p-value[2)] (intergroup)	-0.02 ± 1.63	0.04 ± 1.63	0.861
p -value	0.950	0.881	-
Hb g/L			
Baseline	118.36 ± 13.45	113.58 ± 14.80	0.112
8 weeks	108.96 ± 10.51	111.24 ± 10.70	0.311
Changes from baseline p-value[2)] (intergroup)	-9.40 ± 13.83	-2.33 ± 15.66	< 0.05
p-value	0.0004	0.392	-
N 10^9/L			
Baseline	3.04 ± 0.92	2.99 ± 1.09	0.814
8 weeks	2.88 ± 1.03	3.03 ± 0.88	0.460
Changes from baseline p-value[2)] (intergroup)	-0.15 ± 0.87	0.04 ± 1.17	0.384
p-value	0.440	0.850	-
LYC 10^9/L			
Baseline	1.75 ± 0.49	1.79 ± 0.50	0.702
8 weeks	1.93 ± 0.66	1.77 ± 0.46	0.186
Changes from baseline p-value[2)] (intergroup)	0.17 ± 0.50	-0.03 ± 0.57	0.081

p-value	0.145	0.844	-

Values are presented as Mean ± SD

Table S2 Detailed information on key serum metabolites.

Name	Placebo (mmol/L)	BC99 (mmol/L)	P-Value	Fold_Change
SM(d18:1/17:0)	0.742239281	0.951612215	6.40053E-05	1.282082798
Serine	0.088067827	0.102110309	0.049857312	1.159450766
Taurine	13.32113198	10.72123436	0.035228335	0.804829077
10,13-dimethyl-17-oxo-3-sulfooxy-1,2,3,4,7,8,9,11,12,14,15,16-dodecahydrocyclopenta[a]phenanthrene	100.3616118	175.9393966	0.000478246	1.753054714
Cholesteryl sulfate	31.84892802	41.40484571	0.002924911	1.300038911
Terephthalic-Acid	1.775294478	1.902958717	0.001153805	1.071911585
LacCer(d18:1/16:0)	0.122771012	0.158293353	0.016670665	1.289338183
2-Hydroxy-3-methylbutyric acid	16.11726352	22.36799966	0.009080584	1.387828624
Pregnenolone sulfate	3.046675774	4.681343249	0.010625729	1.536541331
2-Hydroxy-2-methylbutyric acid	16.11726352	22.36799966	0.009080584	1.387828624
Propionylcarnitine (Car(3:0))	2.133506689	1.728022928	0.041080874	0.809944931
Leu-Leu	0.25451767	0.163875867	0.001192282	0.643868328
PC(16:0/18:1(9Z))	0.376636867	0.486739436	0.000600685	1.292330832
Ile-Leu	0.25451767	0.163875867	0.001192282	0.643868328
gamma-Glutamylglutamine	0.06743365	0.085844977	0.026167786	1.273028786
Palmitoyl sphingomyelin	0.69217779	0.84193042	0.008271036	1.216349949
Leu-Ile	0.25451767	0.163875867	0.001192282	0.643868328
SM(d18:1/18:0)	4.869229947	6.009299497	0.000147047	1.234137546
Bisindolylmaleimide I	1.255665332	1.952736326	0.013990559	1.55514075
Cysteine-glutathione disulfide	0.034391522	0.060420081	0.030100232	1.756830694
Palmitoylcarnitine (Car(16:0))	0.360292025	0.435966441	0.012301695	1.210036336
11-Dehydrocorticosterone	24.13093251	28.93284209	0.031203399	1.19899395
Docebenone	24.13093251	28.93284209	0.031203399	1.19899395
PC(18:0/18:1(9Z))	0.734778884	0.846345905	0.027994928	1.151837545
Bilirubin	1.997218163	3.769296093	0.024875951	1.887273089
1,3,7-Trimethyluric acid	0.248854949	0.438562495	0.040851917	1.762321775
SM(d34:1)	5.511505101	6.792392602	0.012993879	1.232402489
Tetraethylammonium cation	0.096973399	0.1406167	0.003368125	1.450054364
1-O-Hexadecyl-2-O-(5Z,8Z,11Z,14Z,17Z-eicosapentaenoyl)-sn-glyceryl-3-phosphorylcholine	1.745862471	2.000024151	0.038493938	1.145579439
1-Palmitoyl-2-docosahexaenoyl-sn-glycero-3-phosphocholine	2.416800997	3.108853042	0.001011191	1.286350447
LPC(22:6)	1.236959447	1.529370013	0.008267073	1.236394626
PC(37:5)	0.138965062	0.178820137	0.016759928	1.286799245
PC(38:5)	0.273072761	0.335510644	0.003879917	1.228649251
Ethyl hydrazinoacetate	1.092522113	1.459107084	0.025667621	1.335540093
L-Isoserine	1.082753641	1.292653411	0.018454651	1.193857367
Androsterone glucuronide	0.127797145	0.226700655	0.003390105	1.773910173

N-Oleoyl-D-erythro-sphingosylphosphorylcholine	6.926964148	7.894392248	0.017372909	1.139661196
N-Methyltrimethylacetamide	0.032830067	0.025317717	0.023472423	0.771174692
1-(1Z-Octadecenyl)-2-(4Z,7Z,10Z,13Z,16Z,19Z-docosahexaenoyl)-sn-glycero-3-phosphocholine	0.415952657	0.491692654	0.01340437	1.182088023
(3.beta.)-Allopregnanolone sulfate	16.70306517	24.70839395	0.027264236	1.479273038
Androsterone sulfate	72.68440394	126.7977159	0.001436613	1.74449688
S1P(d18:1)	0.01985084	0.026617525	0.021407263	1.340876479
LPC(32:0)	0.237167899	0.275456202	0.032943651	1.161439651
(2-{[3-hydroxy-2-tetradecanamidooctadec-4-en-1-yl phosphonato]oxy}ethyl)trimethylazanium	1.491242774	1.924034081	0.011965619	1.290221897
9-Hydroxyrisperidone	0.546103592	0.319087043	0.00530748	0.584297645
Isorhamnetin	0.261166456	0.433599945	0.046371093	1.660243631
Ala-Asp	0.102903069	0.138730466	0.045741868	1.34816646
2-Hydroxy-1,3-dimethyl-9H-thioxanthen-9-one	0.603257701	1.396076501	0.033913555	2.314229059
Fagomine	0.104467133	0.081874231	0.013905778	0.783731967
Citalopram_aldehyde	0.003688522	0.006469636	0.009465163	1.753991426
Dicyclanil	0.212240965	0.298297056	0.041037703	1.405464093
Gln-Glu	0.121790332	0.178362246	0.015525848	1.464502502
PC(18:1(9Z)/P-16:0)	1.059774376	1.356185814	0.019184302	1.279692966
8-Geranyl-7-hydroxycoumarin	6.310382077	7.809693814	0.030192984	1.237594447
Triptophenolide	19.15298073	23.49862096	0.029706772	1.226891067
Amisulpride	20.77930836	37.61156214	0.000368591	1.810048799
(2-aminoethoxy)[2-[docosa-4.7.10.13.16.19-hexaenoyloxy]-3-[hexadec-1-en-1-yloxy]propoxy]phosphinic acid	0.73015273	0.896299532	0.043108813	1.227550751
Simazine	0.144782912	0.236973776	0.04361012	1.636752385
Nilutamide	0.028101684	0.047079502	0.046964382	1.675326731
2-(2,4-Dichlorophenyl)-3-[4-(dimethylamino)phenyl] acrylonitrile	0.703606344	0.923714001	0.03648771	1.312827846
1H-Indole-5-carboxylic acid, 3-(2-methyl-1-oxopropyl)-1-[2-oxo-3-(4-phenoxyphenoxy)propyl]-	0.001131142	0.001902967	0.009335981	1.682340843
PC(18:2(9Z,12Z)/P-16:0)	0.854448654	1.043997284	0.002483085	1.221837356
PC(16:0/P-16:0)	0.18123195	0.234145837	0.00391687	1.291967761
(3beta,23E)-3-Hydroxy-27-norcycloart-23-en-25-one	0.020308414	0.025292291	0.047077161	1.245409478
PC(22:4(7Z,10Z,13Z,16Z)/P-18:0)	0.367979201	0.424083155	0.007658716	1.152465015
(3beta,5alpha,6beta,22E,24R)-23-Methylergosta-7,22-diene-3,5,6-triol	0.274844995	0.33114366	0.006723937	1.204837874
SM(d18:1/20:0)	1.925102472	2.410947699	0.01067623	1.252373697
SM(d16:1/24:1(15Z))	4.957594066	6.46478585	0.003246179	1.30401678
SM(d17:1/24:1(15Z))	1.065118589	1.442863142	0.000582346	1.35465023
PC(22:5(4Z,7Z,10Z,13Z,16Z)/P-18:0)	0.118181388	0.133622565	0.02757674	1.130656593
4-tert-Butyl-N-(4-nitrophenyl)benzamide	0.088456654	0.168308146	0.012813981	1.902718879
[2-[docosa-4.7.10.13.16.19-hexaenoyloxy]-3-(octade	0.785808119	1.040045936	0.003301592	1.323536765

canoyloxy)propoxy]({[2.3.4.5.6-pentahydroxycyclohexyl]oxy})phosphinic acid				
CGS 15943	0.076907555	0.134708646	0.037262938	1.751565835
7-Chloro-8-methyl-2-(2-pyridinyl)-4-quinolinecarboxylic acid	0.003823281	0.005910766	0.006940022	1.545993263
Benzophenone-4	0.519634415	0.727632087	0.017929238	1.400276936
2-[2-(3,4-Dihydroxyphenyl)-2-oxoethyl]thio-4-(2-furyl)-6-methylnicotinonitrile	0.098608872	0.082563883	0.034564174	0.837286551
PHOSALONE	0.059081088	0.085264	0.037584031	1.443169085
SM(d18:0/20:0)	0.189108498	0.239237741	0.023455876	1.265081916
1-Phenyl-1,3-nonadecanedione	0.028676619	0.035866787	0.030647638	1.250732761
Sulindac sulfone	0.082130983	0.092796646	0.036641925	1.129861615
SM(d18:0/16:0)	6.814263906	8.141992599	2.81175E-06	1.194845505
Sennoside	0.056794137	0.116706922	0.027502262	2.054911441
Antibiotic OM 173.alpha.2	0.218092011	0.32072813	0.004367432	1.470609255
[2-(hexadecanoyloxy)-3-[octadec-11-enoyloxy]propoxy]({[2.3.4.5.6-pentahydroxycyclohexyl]oxy})phosphinic acid	0.137721856	0.172772748	0.005943317	1.254504928
Glycyl-Gamma-glutamate	0.054794526	0.066882619	0.02901541	1.220607679
Succinylcarnitine	0.074981582	0.061257857	0.029117618	0.816972065
PC ae (16:0/22:6)	0.142607102	0.23108738	0.009259056	1.62044791
N-[2-Amino-2-carboxyethyl]-glutamate	0.780312298	1.410808986	0.016584577	1.808005575
5-oxo-prolyl-glycine	0.251019454	0.36903229	0.033318557	1.470134224
1-O-Hexadecyl-lyso-sn-glycero-3-phosphocholine	0.30748315	0.376117483	0.04992059	1.223213314
Diacetyl	0.044214352	0.041345343	0.04845902	0.935111375
phosphatidylserine (dioctadecanoyl, n-C18:0)	0.904048332	1.028644684	0.004898445	1.137820454
Dioleoylphosphatidyl-ddc	0.08136662	0.127195734	0.017310359	1.563242194
